# Combover/CG10732, a Novel PCP Effector for *Drosophila* Wing Hair Formation

**DOI:** 10.1371/journal.pone.0107311

**Published:** 2014-09-10

**Authors:** Jeremy K. Fagan, Gretchen Dollar, Qiuheng Lu, Austen Barnett, Joaquin Pechuan Jorge, Andreas Schlosser, Cathie Pfleger, Paul Adler, Andreas Jenny

**Affiliations:** 1 Department of Developmental and Molecular Biology and Department of Genetics, Albert Einstein College of Medicine, New York, New York, United States of America; 2 Department of Biology, University of Virginia, Charlottesville, Virginia, United States of America; 3 Department of Systems & Computational Biology, Albert Einstein College of Medicine, New York, New York, United States of America; 4 Rudolf Virchow Center for Experimental Biomedicine, University of Wuerzburg, Wuerzburg, Germany; 5 Department of Oncological Sciences, The Icahn School of Medicine at Mount Sinai, New York, New York, United States of America; Simon Fraser University, Canada

## Abstract

The polarization of cells is essential for the proper functioning of most organs. Planar Cell Polarity (PCP), the polarization within the plane of an epithelium, is perpendicular to apical-basal polarity and established by the non-canonical Wnt/Fz-PCP signaling pathway. Within each tissue, downstream PCP effectors link the signal to tissue specific readouts such as stereocilia orientation in the inner ear and hair follicle orientation in vertebrates or the polarization of ommatidia and wing hairs in *Drosophila melanogaster*. Specific PCP effectors in the wing such as Multiple wing hairs (Mwh) and Rho Kinase (Rok) are required to position the hair at the correct position and to prevent ectopic actin hairs. In a genome-wide screen *in vitro*, we identified Combover (Cmb)/CG10732 as a novel Rho kinase substrate. Overexpression of Cmb causes the formation of a multiple hair cell phenotype (MHC), similar to loss of *rok* and *mwh*. This MHC phenotype is dominantly enhanced by removal of *rok* or of other members of the PCP effector gene family. Furthermore, we show that Cmb physically interacts with Mwh, and *cmb* null mutants suppress the MHC phenotype of *mwh* alleles. Our data indicate that Cmb is a novel PCP effector that promotes to wing hair formation, a function that is antagonized by Mwh.

## Introduction

Planar Cell Polarity (PCP), the polarity within the epithelial plane, is a characteristic of many epithelia in vertebrates and invertebrates and is established under the control of the non-canonical Wnt/Frizzled (Fz)-PCP signaling pathway. In vertebrates, PCP signaling is evident in the alignment of hair follicles [1], [2] and stereocilia in the inner ear [3], and required for limb growth [4]. Non-canonical Wnt signaling also regulates directional cell migration and intercalation during convergence and extension (C&E) during vertebrate gastrulation and kidney development [5]–[8] and aberrant PCP signaling thus can lead to severe birth defects (reviewed in [9], [10]). In *Drosophila*, PCP signaling controls cell fates and orientation of ommatidia in the facet eye as well as the formation and orientation wing hairs (trichomes; reviewed in [11]–[14]).

A set of core PCP factors including the transmembrane proteins Fz, Flamingo (Fmi; aka. Stan), Van-Gogh (Vang, aka. Stbm), the adaptor proteins Dishevelled (Dsh), and Prickle are required for PCP establishment in all tissues (reviewed in [11]–[13]). Their interplay during PCP establishment leads to their asymmetric localization within cells with Fz and Dsh localizing to the distal and Vang and Pk localizing to opposite proximal vertex of hexagonal wing cells. These asymmetries are thought to act as cues interpreted by downstream effector genes for the establishment of polarity dependent structures [11]–[13]. In particular, each wing cell initiates the growth of a single trichome, an actin and tubulin rich wing hair [15], [16] at the distal vertex at around 30 hrs after puparium formation (APF) [15]–[18]. About 17 hrs later, the trichome has developed into a cuticle ensheathed, rose-thorn shaped spike filled with a highly organized actin and microtubule fibers [19] that points towards the distal wing tip. In core PCP mutants, a wing hair typically forms in the center of a cell and shows aberrant polarity.

Planar cell polarity effector (PPE) genes, such as *inturned* (*in*), *fuzzy* (*fy*), and *fritz* (*frtz*), act downstream of the core PCP genes. In contrast to core PCP mutants, in PPE mutant wings, an average of two independent trichomes are initiated at various positions in the apical periphery of a wing cell (‘multiple hair cell’ (MHC) phenotype) [18]. A distinct phenotype with four hairs per cell is seen in *multiple wing hair* (*mwh*) mutants, some of which appear to be smaller secondary hairs splitting from larger ones [18]. Epistasis analyses and colocalization studies suggest that a complex of In, Frtz, and Fy localizes to proximal, apical cell vertices in a core PCP gene dependent manner and prevents local hair initiation and/or promotes distal hair initiation [18], [20], [21]. Specifically, PCP effector genes recruit and/or activate Mwh via direct interaction with In [22] leading to proximal enrichment of Mwh trailing off towards the distal end of cells [21], [23]. *mwh* encodes a protein that resembles formins in that it contains a Rho family GTPase binding domain followed by a formin homology 3 domain with a potential for dimerization, but lacks a FH2 domain able to catalyze actin polymerization. Mwh may inhibit ectopic actin filament formation either directly, or by interfering with Rho GTPase activation of formins, or formin mediated actin polymerization [21], [23]. Consistent with this, growing actin pimples are initially seen all over the apical surface of a *mwh* mutant wing cell [21]. At around 34 hrs APF, Mwh relocalizes to the base of the forming prehair, where it prevents the formation of secondary trichomes [23].

Fz-PCP signaling also leads to the activation of Rho family GTPases such as RhoA, which in turn activates Rho kinase (Rok) to ensure proper cytoskeletal responses required for trichome formation in the wing and ommatidial rotation in the eye in *Drosophila* or directed cell migration during C&E in vertebrates [24]–[26]. In particular, loss of *rok* causes the appearance of multiple hairs per cell, albeit these trichomes still form at distal vertices and their appearance is thus mechanistically distinct from the action of other PPE genes such as *fy* or *in* [26]. The best-known substrate of Rok is Myosin II light chain regulatory kinase (MRLC, *sqh*), phosphorylation of which is required for myosin activity. Indeed, based on genetic interaction assays, it has been postulated that a proper balance between actin/myosin activities is essential for the formation of a single wing hair, as Myosin II can affect actin bundling [26].

To date, it is unknown how the In/Fy and Mwh PCP effectors cooperate with Rok during wing hair formation. We thus performed a genome-wide molecular screen for novel Rok substrates and identified CG10732 (now called Combover; Cmb) as a novel substrate of Rok. Overexpression of Cmb causes the formation of MHCs, a phenotype that was dominantly enhanced by removal of a gene copy of *rok*. In addition, the MHC phenotype of Cmb overexpression is enhanced by the *fy*/*in* group of PPE genes and *mwh*. We show that Cmb binds to Mwh and that mutation of *cmb* suppresses *mwh* in double mutants. We propose that Cmb is a novel PCP effector, the first one known to act downstream of *mwh* in wing cells during trichome formation.

## Results

### Combover (Cmb)/CG10732 is a novel direct substrate of Rok

**Figure 1:**
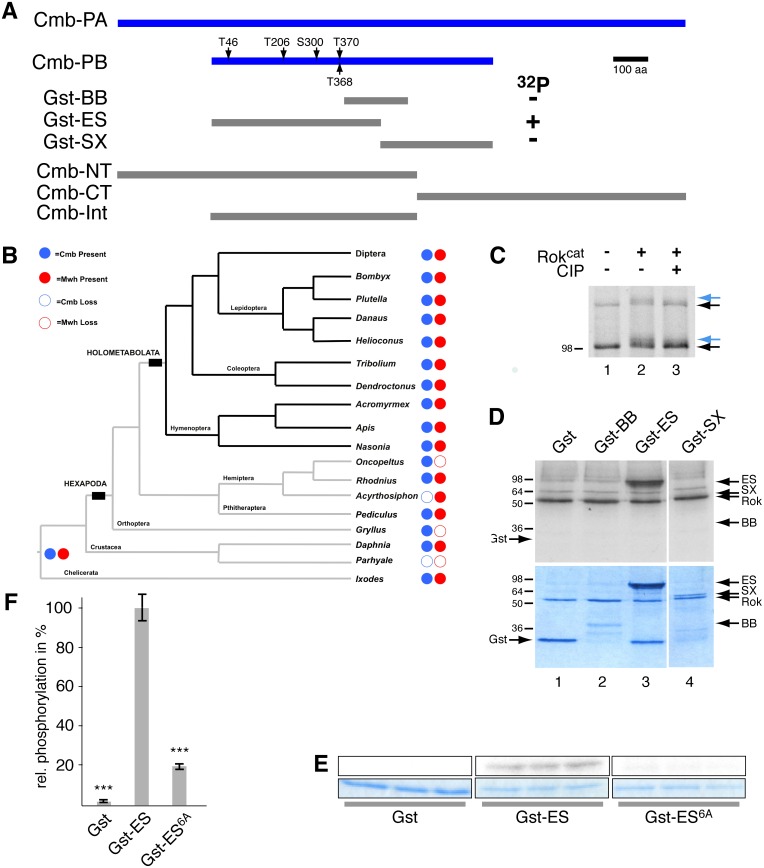
Cmb is a Rho kinase substrate. (A) Schematic of the PA and PB Cmb isoforms as annotated in Flybase. Clone GH01088 is a full-length clone corresponding to *cmb-RB* identified in the Rok target screen. Grey bars indicate GST fusions of Cmb-PB used to map phosphorylation sites in direct kinase assays *in vitro,* and Cmb-NT, Cmb-CT, and Cmb-Int indicate fragments used in two-hybrid and co-immunoprecipitation experiments. Phosphorylation sites identified by mass-spectrometry are numbered relative to Cmb-PB. Direct phosphorylation results of the Cmb Gst fragments are indicated towards the right. (B) Summary of the presence and/or absence of *cmb* and *mwh* genes in model arthropod genomes (see also Figure S2; phylogenetic reconstruction shown is based on [62]–[64]). *mwh* and *cmb* likely evolved in the last common ancestor of euarthropods (as represented by an ortholog in the tick *Ixodes scapularis*). The Diptera clade has been collapsed (see Figures S1 and S2). (C) Treatment of *in vitro* translated Cmb-PB (clone GH01088) with the catalytic fragment of Rho kinase (Rok^cat^) causes the formation of a slower migrating form of Cmb on an Anderson gel (compare blue arrow position with black one in lanes 1 and 2). The gel shift is due to phosphorylation, as it is reverted upon treatment with alkaline phosphatase (CIP; lane 3). Note that for unknown reasons (such as translation initiating at an internal methionine), two Cmb bands are seen upon *in vitro* translation of Cmb in reticulocyte lysates. (D) Phosphorylation of Cmb by Rok is direct. Indicated purified fragments of Cmb fused to Gst were incubated with Rok^cat^ in the presence of [32] P-γATP and separated on a 12% SDS PA gel. Only GST-ES (lane 3) is a substrate of Rok. Top panel is an autoradiograph of the Coomassie stained gel in the lower panel. Arrow show Rok^cat^ autophosphorylation (Rok) and indicated GST-fusion proteins. (E) Kinase assays in triplicate of indicated Gst fusion proteins. The upper panels show autoradiographs of the Coomassie stained gel below. In Gst-ES^6A^, the five phosphorylation sites (see A) identified by mass-spectrometry were mutated to Ala (in addition, T372 was mutated as well, as it lies with in a [TP]_3_ repeat with T368 and T370). (F) Quantification of kinase assay shown in E. Error bars indicate standard deviation; T-test: ***p<0.0001.

To identify novel effectors of Planar Cell Polarity signaling, we performed a genome-wide, gel-shift based screen for Rho kinase substrates [27], [28]. Briefly, pools cDNA clones of the Drosophila Gene Collections 1&2 were *in vitro* translated and internally labeled with [35]S-Methionine and incubated with the catalytic fragment of Rok (Rok^cat^) in the presence of unlabeled ATP. Candidate substrates were identified based on a reduced mobility on Anderson gels [28]. Compared to untreated control, incubation with Rok^cat^ induced a gel-shift of *in vitro* translated clone GH01088 coding for CG10732-PB (Fig. 1A; blue arrows in Fig. 1C indicate the Rok dependent slow-migrating form of CG10732-PB). Importantly, incubation of the kinase reaction with calf intestinal alkaline phosphatase (CIP) strongly reduced the gel-shift (Fig. 1C, lane 3), indicating that the retarded gel migration is dependent on a (direct or indirect) phosphorylation event. CG10732 is thus a novel and uncharacterized potential target of Rho kinase phosphorylation.

Searches in Flybase [29] showed that CG10732, which we named Combover (Cmb), encodes four predicted splice isoforms (*RA-RD*). The open reading frame of the short isoform (PB) being contained within the longest one (PA; Fig. 1A). Isoforms *cmb-RC* and *cmb-RD* lack 96 bp or 99 bp towards the end of Exon 5 of *cmb-RA* (corresponding to Exon 4 of *cmb-RB*), due to removal of an extra intron possibly leading to in frame deletions of 32 and 33 aa, respectively. In contrast to the existence of *cmb-RA* and *cmb-RB* that is strongly supported by genomic data, the existence of *cmb-RC* and *cmb-RD* is only moderately supported [29]. Neither isoform contains known protein domains beyond a potential coiled-coil domain similar to SMC (structural maintenance of chromosomes) proteins (not shown) [30]. To assess when *cmb* evolved, we performed a database search using the BLAST algorithm against model metazoan sequences (see Fig. S1). *cmb* is present across a wide range of insect orders, with the only notable absence in the pea aphid *Acyrthosiphon pisum* (Fig. 1B and S2; alignments in Figs. S3 and S4). We identified a *cmb* ortholog in the crustacean *Daphnia magna* as well as in the tick *Ixodes scapularis.* We did not retrieve orthologs of *cmb* in any non-arthropod metazoan, thus suggesting that *cmb* evolved in the common ancestor of the Euarthropoda (*i.e.* chelicerates, myriapods, crustaceans and hexapods). Phylogenetic analyses revealed that the *cmb* gene exists as a single ortholog in many dipterans (*i.e.* flies and mosquitoes), however many culicomorph ( = mosquito) species have duplicated *cmb* paralogs, including up to three distinct paralogs in *C. quinquefasciatus* and *A. aegypti* (Fig. S2A and B). Our analysis revealed that *cmb* has been maintained in the genomes of a wide range of dipterans, and that *cmb* evolved in the last common ancestor of all arthropod clades.

We then confirmed that Cmb was a direct substrate of Rok *in vitro*. As full-length Cmb-PB was not soluble, we expressed overlapping GST-Cmb fragments (Fig. 1A) in *E. coli* and tested purified fusion proteins in kinase assays in the presence of [32P]γATP. Gst alone, Gst-BB (aa 384–580 relative to Cmb-PB), and Gst-SX (aa 496 to stop) were not phosphorylated by Rok^cat^ (Fig. 1D, lanes 1, 2, 4; note autophosphorylation of Rok^cat^ [31]). However, the N-terminal ES fragment of Cmb-PB (aa 1–495) was directly phosphorylated by Rok^cat^ (Fig. 1D, lane 4), leading to the prediction that the Rok phosphorylation site(s) lie within the first 382 amino acids of Cmb-PB (Fig. 1A). Gst-ES thus was thus subject to phosphorylation by cold ATP and the phosphorylation sites mapped by mass spectrometry. Relative to the start codon of Cmb-PB, T46, T026, S300, T368, and T370 were identified with T368 being phosphorylated at very low levels. Except for S300, all sites are followed by Proline, uncommon for Rok phosphorylation sites that are usually preceded by a positively charged amino acids at position [–1] or [–2] (see [32]–[36]). We mutated these five candidate sites to Ala and introduced an additional mutation (T372A) as it is a third Thr in a [TP]_3_ repeat together with T368, and T370. The mutated Gst-ES^6A^ fusion protein was then tested in direct kinase assays. Fig. 1E shows that compared to Gst-ES (middle set), most phosphorylation of the phosphorylation by Rok^cat^ is lost in the Gst-ES^6A^ mutant (right panel; Fig. 1F shows quantification of the triplicates shown in Fig. 1E) indicating that the major phosphorylation sites of Cmb were correctly identified and that Cmb is a novel Rok substrate *in vitro*.

### A role of Combover in actin wing hair formation

**Figure 2:**
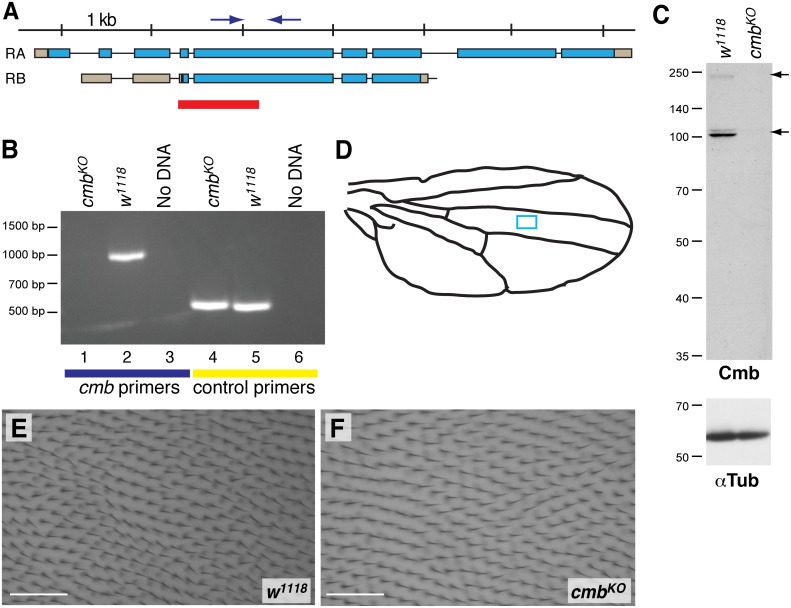
*cmb^KO^* is a protein null mutant. (A) Genomic locus of *cmb/CG10732* showing the *RA* and *RB* isoforms that are well supported by genomic data. 1023 bp of genomic DNA (red) was replaced with a White^+^ marker by homologous recombination to generate the *cmb^KO^* allele. The deleted fragment includes the start codon of the PB isoform. Arrows indicate approximate location of the PCR primers used to verify the deletion. (B) Analytical PCR shows that *cmb* specific primers amplify a 945 bp fragment from *w^1118^* control DNA (lane 2), but not from homozygous *cmb^KO^* DNA (lane 1). Control primers amplify the expected 532 bp fragment from both DNAs showing their integrity (lanes 4, 5). Lanes 3, 6: No DNA controls. (C) Western blot analysis of 3^rd^ instar larval lysates separated on a 12% SDS-PA gel shows that, in contrast to a *w^1118^* lysate (left lane), neither Cmb-PA nor Cmb-PB (arrows; predicted MWs 189 kDa and 89 kDa, respectively) are detected in lysates of homozygous *cmb^KO^* flies. The minor form running above Cmb-PB may be a modified form and was not detected in all preparations. αTubulin was used as loading control (lower panel). (D–F) Wing hairs and their orientation of *cmb^KO^* flies are normal. Compare enlarged wing area of a *w^1118^* wing (E) with a *cmb^KO^* wing in (F; area corresponds to blue box in D). Scale bar is 50 µm.

In order to assess the physiological role of *combover*, we mutagenized the gene using homologous recombination techniques [37], [38]. Briefly, we replaced 1023 bp of Exon 3 including the start codon and roughly half of Exon 4 of *cmb-RB* (equivalent to Exon 4 and half of Exon 5 of the other isoforms; Fig. 2A) with a *white* gene marker. The allele obtained was homozygous viable (see below). The mutation was verified by inverse PCR using primers of the White cassette and genomic primers outside of the homology arms (not shown) and by directly assessing the deletion with primers specific for *cmb* (see Fig. 2A). While cmb primers were able to amplify the expected fragment of 945 bp from wild-type (*w^1118^*) genomic DNA, no product was obtained from DNA of homozygous *cmb^KO^* flies (compare lanes 1 and 2 of Fig. 2B), as predicted for successful deletion. Control amplification of an unrelated locus confirmed the integrity of the DNAs (Fig. 2B, lanes 4, 5).

To further characterize the *cmb^KO^* mutant, we generated an antiserum against the ES fragment of Cmb-PB. *cmb-RA* and *cmb-RB* encode predicted proteins of 1657 amino acids (aa) and 809 aa with calculated molecular masses of 189 kDa and 89 kDa, respectively. Western blot analysis of lysates of *w^1118^* 3^rd^ instar larvae revealed two predominant proteins recognized by the antiserum of about 190 and 110 kDa (as calculated by their relative migration to markers) that were absent from *cmb^KO^* lysates (Fig. 2C; αTubulin was used as loading control, lower panel). Although the smaller isoform migrates at a higher apparent molecular mass than predicted by conceptual translation, the absence of both bands in the mutant show that the *cmb^KO^* allele is a protein null allele of *cmb*.

As *cmb^KO^* flies are viable and showed no gross anatomical defects, we assessed if *cmb* loss caused PCP phenotypes similar to loss of *rok*. Trichome polarity in the wing was normal and we found no multiple hair cell phenotype (compare wild-type wing area shown in Fig. 2E with *cmb^KO^* wing in Fig. 2F). Similarly, sections of adult eyes of homozygous *cmb^KO^* flies were normal, with no PCP defects (Fig. S5).

**Figure 3:**
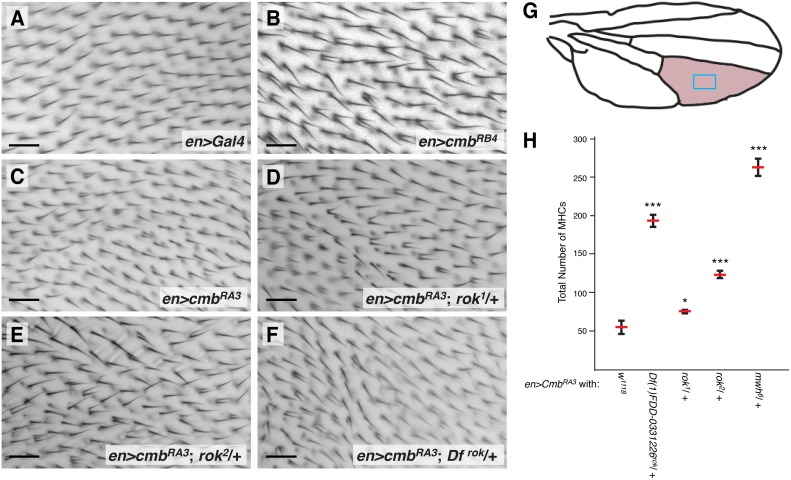
Overexpression of Cmb causes a MHC phenotype which is dominantly enhanced by alleles of *rok*. (A–C) Compared to control (A), overexpression of either isoform of Cmb (RB and RA in (B), (C), respectively) under the control of *en-Gal4* causes the formation of multiple hairs cells specifically in the posterior compartment. (D–F) This MHC phenotype is dominantly enhanced by the removal of one gene dose of rok (*rok^1^* (D), *rok^2^* (E)), or a deficiency uncovering *rok* (*Df(1)FDD-0331226*; F). (G) Schematic of wing indicating the approximate areas shown in panels A–F (blue box) and the second posterior wing cell scored for quantification (rose). (H) Quantification of MHC phenotype of Cmb overexpression in second posterior wing cell and enhancement by indicated alleles. 29°C. Depicted are mean and SEM; T-tests (*p<0.05; ***p<0.001); n≥5. Scale bars are 20 µm.

We next created transgenic flies overexpressing each Cmb isoform under UAS control to assess gain of function (GOF) phenotypes. Overexpression of either Cmb isoform under the control of *sevenless-Gal4*, usually an excellent driver to induce PCP defects in the eye [39]–[41], had no effect (Fig. S5C, D). However, overexpression of *cmb-RA* and *cmb-RB* in several independent transgenic lines under the control of *en-Gal4* (compare *en>Gal4* control wing in Fig. 3A with *en>cmb^RB4^* and *en>cmb^RA3^* in Fig. 3B and C, respectively) or *nubbin-Gal4* (not shown) caused the formation of a multiple hair cell phenotype similar to loss of *rok* (and other PCP effectors) [18], [26]. These data thus suggest a function of Cmb in actin hair initiation or modification of actin myosin contractility. The frequency of ectopic hairs varied strongly between *UAS-Cmb* lines and conditions (not shown), suggesting strong dosage sensitivity.

To assess the specificity of the Cmb GOF phenotype and to assess if there may be a physiological relevance for the *in vitro* phosphorylation of Cmb by Rok, we tested genetic interactions between *rok* and Cmb overexpression. As shown for *rok^1^* and *rok^2^* alleles in Fig. 3D, E, removal of one gene dose of *rok* significantly enhanced the MHC frequency of *cmb-RA* overexpression (quantified in Fig. 3H). Similarly, *cmb-RA* overexpression is enhanced by a deficiency uncovering *rok* (Fig. 3F, quantified in Fig. 3H). Wings of flies heterozygous for *rok* look wild-type (data not shown). These results suggest that rok exerts a negative effect on overexpressed Cmb *in vivo*.

### The multiple hair cell phenotype of Cmb overexpression is enhanced by PPE genes

**Figure 4:**
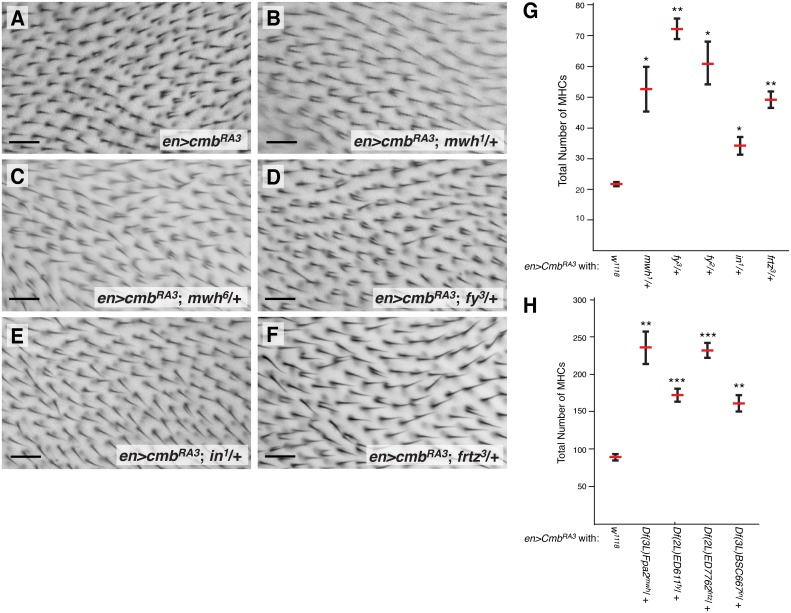
Cmb genetically interacts with PCP effectors. (A) *en>cmb-RA* overexpression phenotype. (B–F) The MHC phenotype of *en>cmb-RA* is dominantly enhanced by the removal of one gene dose of *mwh^1^* (B), *mwh^6^* (C), *fy^3^* (D), *in^1^* (E), and *frtz^3^* (F). (G–H) Quantification of MHC phenotype of *cmb-RA* overexpression in second posterior wing cell (see Fig. 3G for schematic) and enhancement by indicated alleles (G) and deficiencies uncovering those loci (H). Baselines are variable between the different experiments and quantification in (G) corresponds to experimental series shown in B, D–F; quantification of the *mwh^6^* interaction is shown in Fig. 3H. Graphs show means and SEM; T-tests, reduced Bonferroni correction (*p<0.05; **p<0.01; ***p<0.001); n≥5. 29°C. Scale bars are 20 µm.

To further define a potential connection between Cmb and PCP effectors, Cmb was overexpressed in the *Drosophila* wing in a heterozygous mutant background for members of the PCP effector family. Intriguingly, the MHC phenotype of *cmb-RA* overexpression is dominantly and statistically significantly enhanced by removal of one gene dosage of *mwh^1^, mwh^6^, frtz^3^, fy^2^, fy^3^,* or *in^1^* (Fig. 4A–F; quantified in Fig. 4G and 3H) as well as by heterozygosity for deficiencies uncovering each of these loci (quantified in Fig. 4H; control wings of PPE heterozygotes look normal; data not shown). This clearly suggests that while Combover is not essential for trichome formation, nevertheless it can affect actin wing hair formation, possibly being negatively regulated by the planar cell polarity effector protein family.

### Combover physically interacts with and is regulated by the PCP effector Multiple Wing Hairs

**Figure 5:**
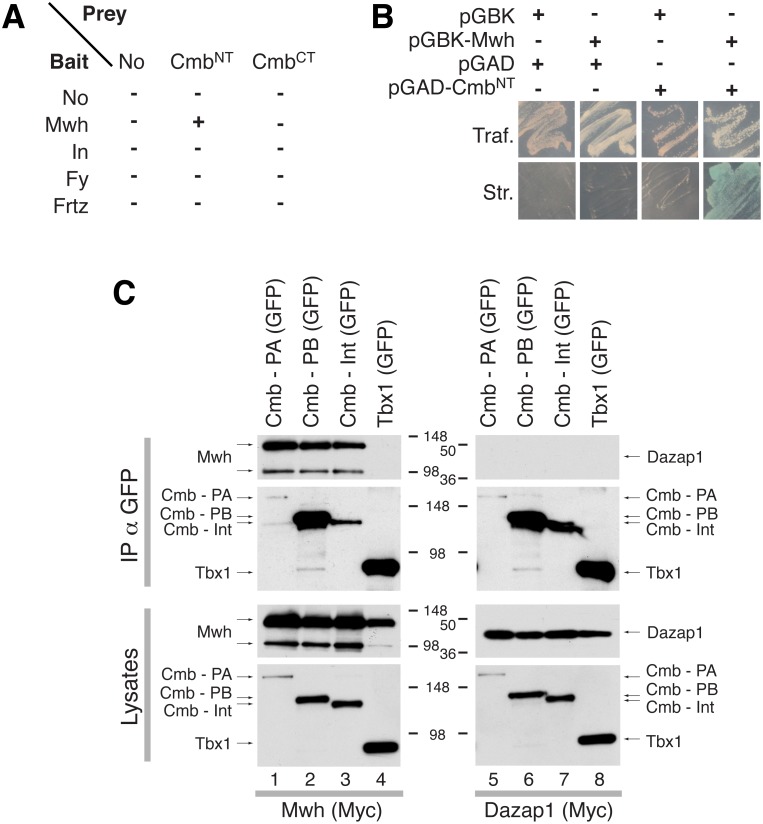
Cmb interacts physically with Mwh. (A) Summary of yeast two-hybrid interaction assays between PCP effector candidates (baits) and Cmb N- and C-terminal parts (Cmb^NT^ and Cmb^CT^, respectively; c.f. Fig. 1A). Only Mwh, but not In, Fy, or Frtz interacts with Cmb. (B) Yeast two-hybrid results of transfections with indicated plasmids. Upper panels show growth under conditions selective for the presence of the plasmids only (Traf.). Lower panels: additional stringent selection for interaction (Str.: -HIS; -ADE) furthermore showing interaction via LacZ staining, a third maker present in the yeast strain. Only yeast cells containing the Mwh bait and the Cmb^NT^ prey, but not the controls grew under conditions selective for interaction. (C) Cmb specifically coimmunoprecipitates Mwh from Lysates of HEK293 cells. Indicated GFP-tagged Cmb constructs (lanes 1–3, and 5–7; see Fig. 1A for schematics) or Tbx1 (lanes 4, 8; negative control) were cotransfected with Myc-tagged Mwh (lanes 1–4) or Myc-Dazap1 (lanes 5–8; negative control) and immunoprecipitated with anti GFP antibodies. Upper panels show immunoprecipitations, lower panels show lysates probed with antibodies recognizing the tags of the indicated proteins. Note that Cmb-PA transfers very inefficiently onto membranes.

To assess the mechanistic basis of the genetic interactions, we tested whether Cmb would physically interact with PPE genes in yeast two-hybrid assays. Due to the size of Cmb, we tested interactions of Fy, Frtz, In, and Mwh bait proteins for interaction with the N- and C-terminal halves of Cmb as prey (see schematic in Fig. 1A). Interestingly, the N-terminal half of Cmb-PA interacted with Mwh in this assay under stringent selection conditions on medium simultaneously lacking Ade and His (Fig. 5A and 5B). In addition, growth correlated with the activation of the *lacZ* gene, a third reporter present in the two-hybrid tester strain. Neither Fy, Frtz, or In interacted with Cmb (Fig. 5A), also suggesting the Cmb-Mwh interaction is specific.

To independently confirm the interaction in a different system, we transfected HEK293 cells with GFP tagged Cmb-PA, Cmb-PB, or Cmb-Int, a fragment consisting of the N-terminal half of Cmb-PA overlapping with Cmb-PB (Fig. 1A) together with Myc-tagged Mwh. Immunoprecipitation with anti-GFP antibodies efficiently co-precipitated Mwh (lanes 1–3 in Fig. 5C; note that Mwh is expressed as a doublet in HEK293 cells), but not a Myc-tagged control protein (Dazap1, lanes 5–7 in Fig. 5C). Importantly, GFP-Tbx1 did not pull-down Myc-Mwh (Fig. 5C Lane 4), indicating that Cmb specifically interacts with Mwh not only in yeast two-hybrid assays, but also in lysates of transfected cells.

**Figure 6:**
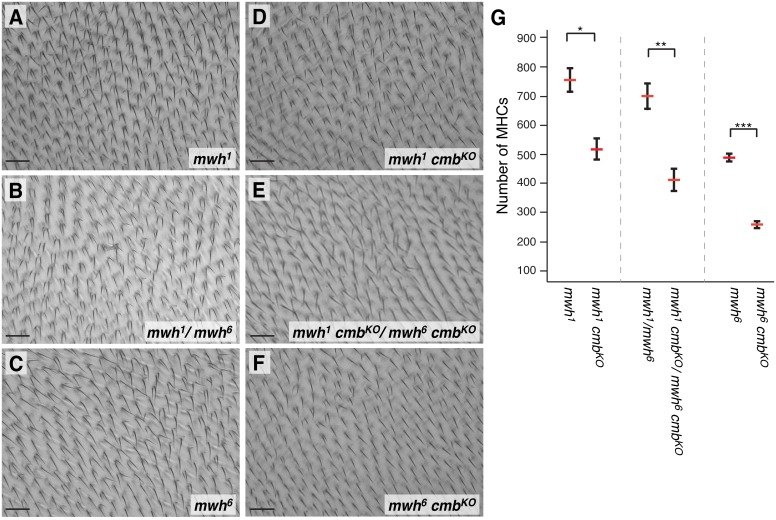
*cmb* suppresses *mwh* in double mutants. The multiple hair cell phenotypes of *mwh^1^* (A), *mwh^1^*/*mwh^6^* transheterozygotes (B), or *mwh^6^* (C) single mutants, are significantly suppressed in corresponding double mutants with *cmb^KO^* (D–F). Wing areas depicted correspond to the blue box in Fig. 3H. (G) Quantification of number of MHCs in the second posterior wing cell (Fig. 3H) of indicated genotypes. Note that all allelic combinations of *mwh* are suppressed by concomitant loss of *cmb*. Graphs show means with SEM; T-tests, Bonferroni correction (*p<0.05; **p<0.01; ***p<0.001); n≥5. Scale bars are 20 µm.

The Fy/In group of PCP effectors form a complex that is required for the proper localization and probably the restriction of Mwh activity to the more proximal side of early developing pupal wing cells [21]–[23]. Furthermore, the MHC phenotype of Cmb overexpression is enhanced by reduction of *fy*, *in*, *frtz*, and *mwh* (Fig. 4). Our physical and genetic interaction data suggested that Cmb contributed to trichome formation, a function that might be antagonized by PPE genes. If so, we hypothesized that loss of *cmb* might suppress the phenotype of *mwh* mutants. We thus recombined the *cmb^KO^* mutation with an amorphic *mwh^1^* and a hypomorphic, temperature sensitive *mwh^6^* allele [18], [23], [42] and assessed the phenotypes of homozygous double mutants raised at 25°C. Indeed, loss of *cmb* suppressed the phenotype of *mwh^1^* and *mwh^6^* alleles, as well as *mwh^1^*/*mwh^6^* transheterozygotes (compare Fig. 6A–C with D–F). Quantification of the multiple hair cells in the second posterior wing cell (Schematic in Fig. 3G) showed that the suppression from a mean of 752±40 MHCs to 517±35 for *mwh^1^*, of 697±44 MHCs to 413±39 for *mwh^1/^mwh^6^*, and of 488±13 MHCs to 260±12 for *mwh^6^*, respectively, is statistically significant (Fig. 6G), thus supporting a model in which Cmb has an role in actin wing hair formation and is directly regulated by the PCP effector Mwh through a protein-protein interaction *in vivo*.

### Localization of Cmb in pupal wing discs

**Figure 7:**
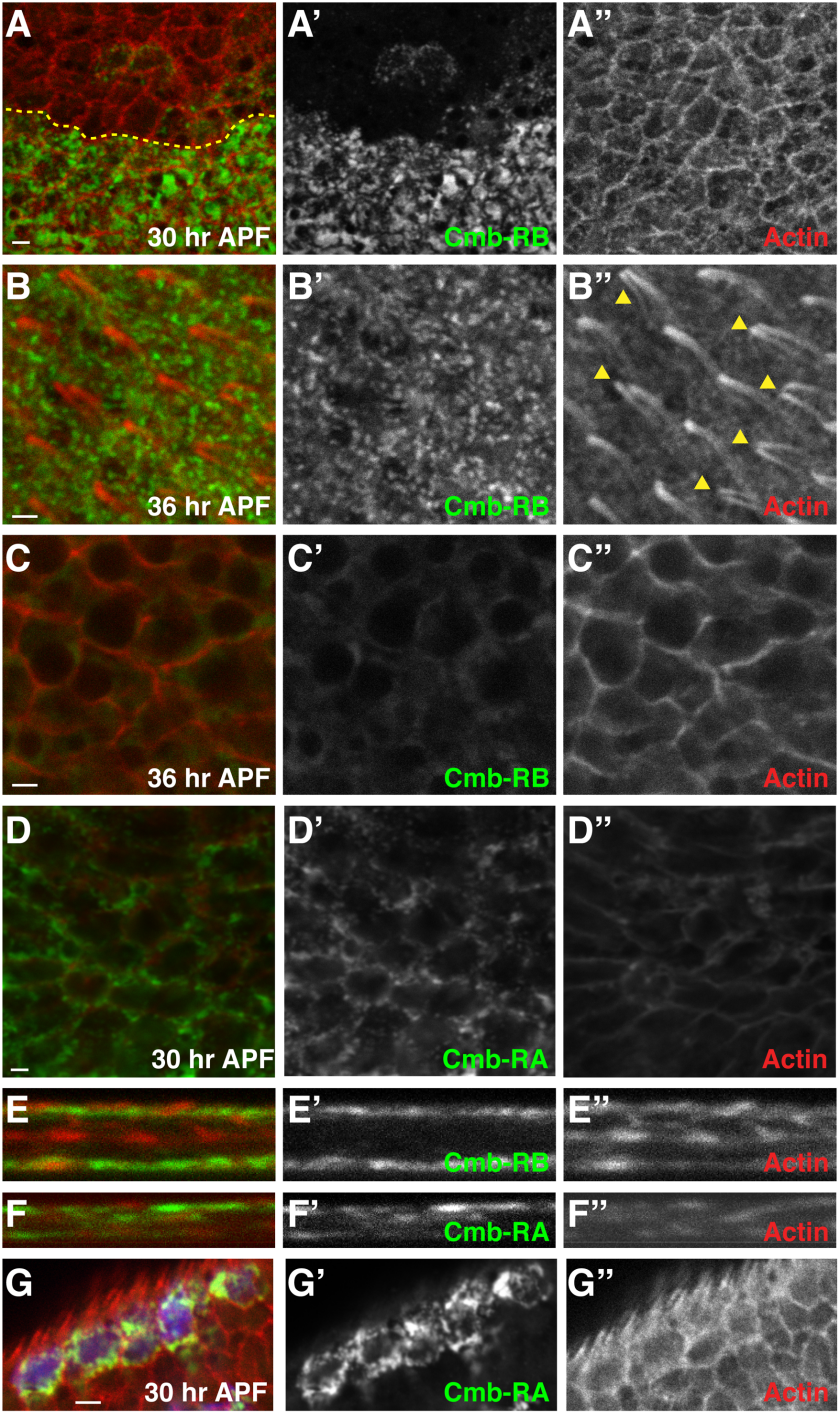
Cmb localization. Pupal wing discs expressing *en>cmb-RB* or *en>cmb-RA* (green) in the posterior compartment (posterior is down, distal to the right in all panels). Actin is shown in red; greyscale pictures show indicated single channels. (A) Endogenous Cmb (anterior, above compartment boundary indicated by a yellow dotted line) is not detected by our antibody. At 30 APF prior to hair initiation, cmb-RB overexpressed in the posterior compartment localizes apically in a punctate pattern. (B–C) At 36 APF, after hair initiation, *en*>*cmb-RB* localizes apically (B, B’) but is largely absent from more basal confocal sections (C, C’). Note the multiple wing hairs due to Cmb overexpression emerge on the distal side (C”). Cmb is not present in the wing hairs. (D) *en>cmb-RA* localizes apically in 30 hr APF wing discs and appears cortically enriched in cells that express at a lower level. (E, F) Optical Z-sections of 36 hrs pupal wing discs shows strong apical enrichment of cmb-RB (E, E’) and cmb-RA (F, F’; note that the dorsal and ventral wing layers are seen, with their basal sides touching and their apical sides facing away from each other, respectively). (G) Flip-out clones (marked by UAS-GFP in blue in G) expressing *cmb-RA* under the control of *actin-Gal4* show that while cmb-RA appears cortically enriched, it is not asymmetric with respect to the P/D axis. Scale bars are 2 µm.

In order to restrict wing hair formation to the distal end of cells, In and Frtz are localized to the apical, proximal side of developing wing cells prior to hair formation in a process that is controlled by the core PCP signaling module [20], [21], [23]. Similarly, Mwh is enriched at the proximal edge of wing cells [21], [23]. We thus stained pupal wing discs with our Cmb antibodies. Unfortunately, our antiserum does not detect endogenous Cmb in tissue samples (Fig. 7A, A’). *en>cmb-RB* and *en>cmb-RA* driven protein is localized apically in posterior wing cells at 30 hrs APF prior to wing hair formation (Fig. 7A, D). We noticed that the expression level of the transgenes was somewhat variable between different cells. In cells expressing Cmb at lower levels, it appears cortically enriched. At 36 hrs APF, once wing hairs started forming, Cmb protein remains distributed in a grainy pattern in the apical region of wing cells (Fig. 7B, B’ for *cmb-RB* and not shown) and is largely excluded from more basal regions of cells (Fig. 7 C for *cmb-RB*). Apical restriction of both isoforms is also evident in Z-sections (Fig. 7E, F). We do not find Cmb localized to the actin hair itself (Fig. 7B). Significantly, the ectopic hairs form at the distal vertex (Fig. 7B) and no obvious difference in Cmb localization is seen between cells that show one or multiple hairs due to overexpression of Cmb (Fig. 7B, B’ and B”). To express Cmb at lower levels and to address if Cmb can localize asymmetrically in cells, we induced ‘flip-out’ clones expressing *cmb-RA* under control of the *actin-Gal4.* While we find apical cortical enrichment of Cmb-RA, there is no evidence for proximal-distal asymmetry (Fig. 7G, G’; expressing cells are labeled in blue in G). In conclusion, (overexpressed) Cmb is localized apically in pupal wing cells and is cortically enriched in cells that appear to express lower protein levels and thus overlaps with areas where Mwh (and other PCP effectors) localize.

## Discussion

Rho kinase, a member of the AGC kinase family which also includes PKC and Akt (for review see [43], [44] was originally identified as a RhoA effector reorganizing the cytoskeleton by promoting the formation of actin stress fibers [45]. In *Drosophila*, Rok was shown to act downstream of Fz and Dsh in the non-canonical Wnt/Planar Cell Polarity pathway causing ommatidial rotation and structural defects in the eye and multiple hairs cells in the wing [26]. Here, we have identified Combover/CG10732 as a novel substrate of Rok. We created a *cmb* protein null allele lacking both Cmb protein isoforms (Fig. 2C) that is homozygous viable. Homozygous *cmb* mutants display no visible phenotype in the wing or in sections of the adult eyes (Figs. 2 and S5). As a reduction or an excess of actin polymerization can cause MHCs, we assessed the overexpression phenotype of Cmb. Indeed, overexpression of either Cmb isoform caused a multiple hair cell phenotype that is strongly dominantly enhanced by *rok* and the *fy/in/mwh* PCP effectors, validating our *in vitro* screening approach to identify PCP effectors. Importantly, the *cmb* mutation suppresses the MHC phenotype of *mwh* in double mutants. Our data thus indicate that Cmb, while not essential for wing hair formation, nevertheless promotes trichome formation *in vivo*.

### Rok phosphorylates unconventional sites on Cmb

It has been noted that known phosphorylation sites of Rok targets such as ERM proteins, Vimentin, Myosin regulatory light chain, or Adducin, often follow the consensus site [R/K]XX[S/T] or [R/K]X[S/T] [32]–[36]. Of the five Rok sites we have identified in our *in vitro* kinase assays followed by MS analysis, only S300 is preceded by a basic residue at position [–2] (RT[S]). In all other cases, no basic amino acid is found at position [–1] or [–2]. However, T46, T206, T368, and T370 are all followed by a Proline, more typical of MAP kinase phosphorylation sites [46]. Nevertheless, mutation of these sites strongly reduced Cmb phosphorylation *in vitro* (Fig. 1E).

**Figure 8:**
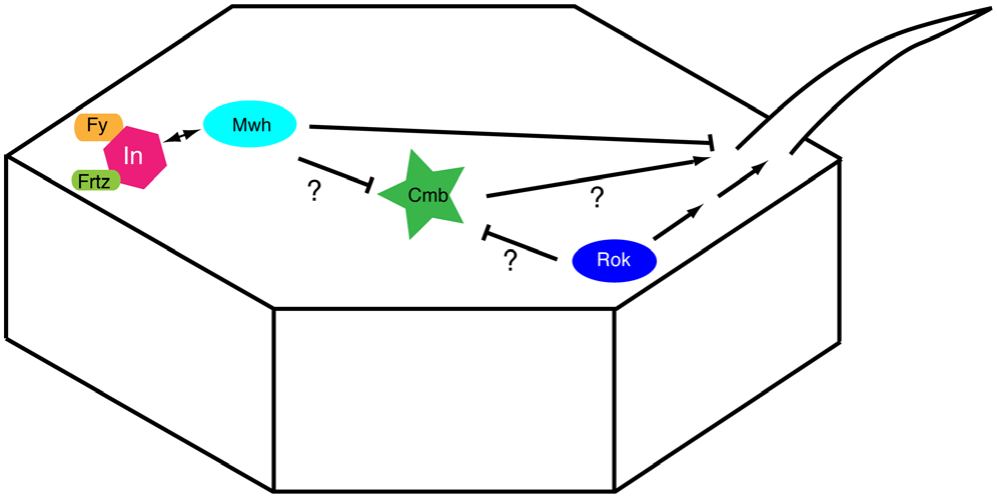
Model of Cmb function. Based on the genetic interaction data we suggest that Rok may antagonize a positive role of Cmb on wing hair formation. Mwh is enriched on the proximal side of wing cells by the Fy/In group of PCP effectors where it prevents hair initiation. The suppression of the MCH phenotype of a *mwh* null allele by a *cmb* null mutant further suggests that the role of Cmb on hair formation is antagonized by Mwh.

In *rok* mutants, multiple hairs form at the distal end of wing cells [26]. Similarly, overexpression of either Cmb isoform causes MHCs that originate at the distal end of cells (Fig. 7B), distinct from the *in/fy* group of PCP effectors and *mwh*, which form MHCs around the periphery of the cells (note that in *mwh* mutants, actin patches are initially even formed all over the apical cell surface) [18], [21]. Importantly, reduction of *rok* activity by the removal of one gene dose (by two different alleles or a deficiency) increases the number of MHCs (Figure 3), suggesting an inhibitory effect of Rok on Cmb. It was suggested that Myosin II, which is concentrated at the site of prehair initiation and whose activity is regulated by Rok via phosphorylation of its regulatory light chain (MRLC), must be within an optimal range to properly bundle actin and to ensure the formation of a single hair [26]. Consistent with the genetic interaction between *cmb* and *rok*, it is possible that in addition to regulating MRLC, Rok might also inhibit a potential hair promoting activity of Cmb (see model in Figure 8), although we cannot exclude that Rok/MRLC activity acts in parallel to the effect Cmb exerts on wing hair formation.

### Cmb as a PCP effector during wing hair formation

*mwh* and the *in*/*fy* group of PCP effectors all have been implicated in restricting actin hair initiation to the distal vertex of the cells by inhibiting proximal hair assembly [18], [20], [21], [23], [47]. Core PCP signaling ensures proper proximal localization of In, Frtz, and Fy proteins (and thus inhibition of prehair formation) to the proximal end of wing cells [18], [21], [23] leading to the formation of a single wing hair on the distal side.

*cmb-RA* or *cmb-RB* cause the formation of MHC phenotypes upon overexpression with several wing drivers. Importantly, this overexpression phenotype is enhanced by the removal of one gene dosage of the PCP effectors *fy*, *frtz*, *in*, and *mwh* as well as deficiencies uncovering those loci. These genetic interactions suggest *cmb* could exert a positive effect on hair initiation, although such a function would play a supportive or redundant role as neither a lack or ectopic trichomes are found in *cmb* mutants.

Significantly, we showed that Cmb physically interacts with Mwh in yeast two-hybrid and coimmunoprecipitation assays (Fig. 5). Interestingly, while we were unable to identify vertebrate homologs of *cmb*, we found orthologs of both *cmb* and *mwh* outside of the insects in the genomes of the crustacean *Daphnia magna* and of the tick *Ixodes scapularis* (Figs. 1B, S1, S2; see also [21], [23]). *Ixodes* is a member of the Chelicerata, the most basally-branching euarthropod clade that split from the remaining arthropod groups in the Cambrian [48]. The presence of *mwh* and *cmb* in *Ixodes* may be indicative of an ancient protein-protein interaction that has been retained throughout arthropod evolution. Because both *Ixodes* and *Daphnia* lack wings, the Mwh/Cmb interaction likely performed different, possibly additional function in the ancestral arthropod. Consistent with this, *mwh* mutants cause other cuticular hair defects in other regions of the *Drosophila* body [49]. Alternatively, the Mwh/Cmb interaction evolved much later than the appearance of both of these genes in the genome of the ancestral arthropod. The roles of and interactions between Cmb and Mwh proteins in more non-insect arthropods needs to be further explored.

The presence of both *mwh* and *cmb* orthologs in the genomes of members of all holometabolous insect orders may indicate that the Mwh and Cmb interaction is also conserved in this insect clade. The retention of these two genes in members of the more basally-branching hemipteran orders, however, is less conserved. The conservation of *mwh* and *cmb* in Holometabolata may be due to their shared mode of wing development, *i.e.* via internal wing imaginal discs. This is in contrast to the mode of wing development in hemimetabolous insects by which the wings develop as buds outside of the body. Further study into the association of wing development and Mwh/Cmb interactions in other insect orders is needed to elucidate these findings.

Interestingly, PCP effector mutations generally enhance each other. For example, the hypomorphic *frtz^3^* allele is enhanced by weak alleles of *in* or *fy* in double mutants [47]. Analogously, removal of a gene dosage of mwh in a *fy* or *in* background, enhances their MHC phenotype [50]. In contrast, the MHC phenotype of *mwh* mutants was (partially) suppressed in *mwh cmb* double mutants, as significantly fewer cells formed additional hairs. This interaction is likely specific, because we find it with the temperature sensitive *mwh^6^* allele [18] and with the spontaneous *mwh^1^* allele, two alleles of independent origin unlikely to carry a similar second site mutation and thus further supporting the physiological function of Cmb as a PCP effector. To our knowledge, *cmb* is the only gene reported so far to suppress *mwh*. Importantly, as *mwh^1^* and *cmb* both are null alleles (*cmb^KO^* lacks expression of both protein isoforms, Fig. 2; [23]), this result suggests that Mwh acts upstream of and normally antagonizes Cmb and that the derepression of Cmb thus may contribute to the MHC phenotype of *mwh* mutants (Fig. 8).

Unfortunately, our Cmb antibodies do not detect endogenous Cmb protein in the developing pupal wing. Nevertheless, Cmb expressed in the posterior compartment of the wing under the control of *en-Gal4* localizes apically in a punctate pattern. In cells that appear to express at a lower level (seen particularly for Cmb-RA in Figure 7D, G) Cmb is enriched at the circumference of the cells, but shows no proximo-distal enrichment. Although we cannot exclude that Cmb localization is an overexpression artifact, this appears unlikely, because we would expect Cmb to fill the cells rather than to localize specifically apically (Fig. 7G). Importantly, Cmb likely localizes to the area of wing cells where Mwh is present, as Mwh known to be initially enriched apically towards the proximal side [21], [23], further supporting our model that a positive effect of Cmb as a novel PCP effector on wing hair formation may be restricted by Mwh.

## Materials and Methods

### Ethics Statement

This study was carried out in accordance with the recommendations in the Guide for the Care and Use of Laboratory Animals of the National Institutes of Health. The antibody generation protocol was approved by the Institutional Animal Care and Use Committee of the Albert Einstein College of Medicine (Protocol number 20130514).

### Fly strains

*mwh^1^*, *mwh^6^*, *in^1^*, *fy^2^*, *fy^3^*, *frtz^3^* are described in Flybase [23], [47], [51], [52]. *Df(3L)Fpa2* (*mwh*), *Df(2L)ED611* (*fy*), *Df(2L)ED7762* (*frtz*), *Df(3L)BSC667* (*in*), *Df(1)FDD-0331226* (*rok*) were obtained from the Bloomington stock center. *rok^1^, Frt19A* and *rok^2^, Frt19a* were a kind gift of Dr. L. Luo (Stanford University). Constructs for transgenic flies were injected by Rainbow Transgenic Flies and Genetic Services.

For adult wing analysis, wings were incubated in 0.1% Triton-X100 in PBS for at least one hour and subsequently mounted in 80% glycerol in 1xPBS. Multiple hair cells (MHCs) were counted on a minimum of 5 wings was utilized. Statistical analyses were performed using R version 3.0.2 (2013-09-25).

### Plasmids

All PCR products used for cloning were sequence verified. pCS3_Myc_Dazap1 was a kind gift of Dr. F. Marlow, Einstein. pCS_Tbx1_GFP was a kind gift of Dr. B. Morrow (Einstein).

pFastBacHisC was constructed by inserting Fast_bac_his_upper and Fast_bac_his_lower into the BamHI/HindIII sites of pFastBac1 (Life Technologies). pCRIITopo_rok was made by cloning the Rok open reading frame as a PCR product amplified with drok_Upper_BglII and drok_lower_BamHI into pCRIITopo (Invitrogen). pFastBacRGSHis_rok^Cat^ was made by cloning a BglII/EcoRI fragment of pCRIITopo_rok into the BamHI/EcoRI sites of pFastBacRGSHis. pFastBacRGSHis_rok^Cat^ was transformed into DH10BAC cells and Baculovirus was produced according to the instructions of the manufacturer (Invitrogen). 1l of SF9 cells (10^6^ cells/ml) was inoculated at a MOI of 1 and Rok^cat^ was expressed for 72 hrs at room temperature in a spinner flask. Cells were incubated in 40 ml lysis buffer (50 mM Tris, 150 mM NaCl, 10% glycerol, 3 mM β-mercaptoethanol, 10 mM imidazole, pH 8.0 supplemented with 1x Complete protease inhibitors (Roche)) for 10 minutes on ice and sonicated. The lysate was centrifuged for 20 min at 10,000 g and the supernatant incubated with 2 ml 50% NiNTA agarose (Qiagen) for 1 hour. After 3 washes with 10 ml lysis buffer, the protein was eluted with 3 ml lysis buffer containing 300 mM imidazole and the eluate dialyzed against lysis buffer (without protease inhibitors and imidazole). Protein concentration was estimated to be 300 ng/µl using BSA standards on a Coomassie stained gel.

To generate the *cmb^KO^* knock-out allele, left and right homology arms were amplified by PCR from Bac clone RP98-17E13 (DGRC, CHORI, CA) using primers CG10732_left_for_KpnI, CG10732_left_rev_SacII and CG10732_right_for_BglII, CG10732_rarmLong_rev_AvrII, respectively and cloned into pSCA_KanAmp (Agilent). After sequence verification, the left arm was cloned as KpnI (blunt)/SacII fragment into the NotI (blunt)/SacII fragment pRK2 [37], followed by insertion of the right arm as BglII/AvrII fragment into the corresponding sites to give pRK2_CG10732_final. The knock-out mutant of *cmb* was made according to [37]. The integrity of the *cmb^KO^* allele was verified by inverse PCR (not shown) and by PCR analysis using primer Cmb KO Verification F and Cmb KO Verification R (predicted product size 945 bp; Fig. 2A). To control for DNA integrity, primers EY10165_1 and EY10165_2 were used to amplify a fragment of CG7177 of 532 bp.

pGEX4T3_ES (Gst-ES) was generated by isolating the EcoRV/Sal fragment from pOT2_CmbRB (DGC clone GH01088) and cloning it into the SmaI/SalI sites of pGEX4T3. pGEX4T2_SX (Gst-SX) was made by isolating the SalI/XhoI fragment of pOT2_CmbRB and cloning it into the SalI site of pGEX4T2. pGEX4T2_BB (Gst-BB) was generated by isolating BamHI fragment (blunt) from pOT2_CmbRB and cloning it into pGEX4T(SmaI) vector.

pSCA-K/A-CmbRA_Nterm and pSCA-K/A-CmbRA_Cterm were made by cloning PCR products amplified from oligo-dT primed total ovarian cDNA using CG10732_RA_For and CG10732_RA_Nrev, and CG10732_RA_Cfor and CG10732_RA_rev, respectively, into pSca-KA (Agilent). pSCA-K/A-CmbRA was then assembled in a triple ligation from pSCA-K/A-CmbRA_Nterm (DraIII/EcoRV), pOT2_CmbRB (EcoRV/DraIII), and pSCA-K/A-CmbRA_Cterm (DraIII fragment; note that the different DraIII sites have different spacer sequences and could thus only ligate in one way). A gateway entry clone for CmbRA was made by inserting a XbaI (blunt)/BglII fragment of pSCA-K/A-CmbRA into the EcoRV/BamHI sites of pENTR_3C. A CmbRB entry clone was generated by cloning a PCR product amplified with CG10732RB_For and CG10732RB_Rev primers into the pCR8 Gateway entry vector. pCS3_GFP and pTFW versions of CmbRA and CmbRB were made by Gateway recombination according to the instructions of the manufacturer (Invitrogen, CA) [53], [54]. pCS2_GFP_Cmb-Int was made by isolating the internal Cmb BspEI fragment from pCS3_GFP RB and inserting it into the BspEI site of pCS2-EGFPC1. pCS2-EGFPC1 was made by cloning EGFPC1 as NheI (blunt)/BamHI (blunt) fragment into the XhoI (blunt)/BamHI (blunt) sites of pCS2(105). pCS2_Myc6_Mwh was made by inserting a NdeI (blunt)/SalI (blunt) fragment of pGBKT-Mwh [22] into the XhoI site (blunt) of pCS-MT.

Site directed mutagenesis of the Rok phosphorylation sites of Cmb (T/S->A) was performed in successive rounds by using pGEX4T3_ES and the QuickChange mutagenesis kit (Agilent) with primers that are detailed below. T46A, T206A, S300A are tagged with CfoI, BsaXI and BfaI sites, respectively. The T368A T370A, T370A cluster is tagged with a MsiI site (note that even though T370 was not identified as a Rok sit *in vitro*, it was mutated, as it corresponds to the last Thr in a [TP]_3_ repeat.

Cmb two-hybrid pGADT7_Cmb^NT^ and pGADT7_Cmb^CT^ prey constructs were made by cloning the N- and C-terminal halves of CmbRA as PCR products amplified from pScaKA_CmbRA with CG10732-NdeI-F1 and CG10732-XmaI-R1, and CG10732-NdeI-F2 and CG10732-XmaI-end-R2, respectively, as NdeI/XmaI fragments into pGADT7. pGBKT constructs of *mwh* and *fy* were made by cloning PCR products amplified with DBD-mwh5 and DBD-mwh3, and pGBKT7-5′Fy and pGBKT7-3′_Fy, respectively, as NdeI/EcoRI fragments into pGBKT7. Analogously, *frtz* was cloned into the EcoRI site of pGBKT7 after amplification using primers 5′frtz-hybrid and 3′fritz-hybrid, and *in* was cloned into NheI/BamHI of pGBKT7 after amplification with Inturn-th5 and Inturn-th3.

### Antibody production and Western analysis

Gst-ES was expressed and purified by the Macromolecular Therapeutics Development Facility of Einstein. Briefly, after purification over a Gst-Sepharose column, Gst was cleaved off with His_6_-tagged Thrombin and further purified over a Ni-column and by size-exclusion chromatography. 100 µg ES fragment was injected per boost into Guinea pigs by Covance. For Western analysis, lysates of 7 3^rd^ instar *w^1118^* and *cmb^KO^* larvae were prepared as the HEK293 cells for Co-IP were and separated on a 12% SDS PAGE gel, transferred to PVDF membrane and probed with #1051 anti Cmb at a dilution of 1∶5,000. Signals were detected with a rabbit anti-Guniea pig antibody (Invitrogen) at a dilution of 1∶10,000 and imaged after ECL treatment (Promega).

### Kinase assays

For gel shift assays, 0.5 µl miniprep DNA of pOT2_CmbRB (DGC clone GH01088) were *in vitro* translated in the presence of [35] S-methionine in a 10 µl reaction using the TNT coupled transcription-translation system (Promega) according to the instructions of the manufacturer. The translation was then diluted with 40 µl 1.25x kinase buffer (5x kinase buffer: 125 mM HEPES pH 7.2, 15 mM MgCl_2_, 5 mM EDTA; unless phosphatase assays were performed, 5x kinase buffer also contained 25 mM β-glycerophosphate and 5 mM Na_3_VO_4_). 5 µl of this dilution was incubated for 1 h with 5 µl 2x kinase mix (1 µl 5x kinase buffer, 1 µl 5 mM ATP. 0.1 µl 100x Cycloheximide (Sigma), 0.5 µl Rok^cat^ and, if required, 10 u calf intestinal alkaline phosphatase (Roche). The reaction was stopped by the addition of 5 µl 5x SDS loading dye and boiling for 5 min at 95°C. Kinase reactions were separated on a 12% Anderson gel [28].

Gst proteins were expressed as described [40], [41]. Radioactive kinase assays consisted of 250 ng of purified GST-tagged protein, 4 ul 5x Kinase buffer, 1.5 ul of 1 mM ATP, 0.5 ul of 32P γATP(3000 ci/mmol), 0.5 ul of Rok^cat^ in a total volume of 20 µl. The assay was incubated at 25°C for 1 hour and the reaction was stopped by the addition of 5 ul of SDS Loading dye followed by denaturation for 5 minutes at 95°C. The assay was then separated on a 12% SDS- PAGE gel. After staining gels with Coomassie Blue, gels were scanned for quantification of total protein amounts, dried and exposed on a Fuji FLA9000 phosphorimager to quantify the extent of phosphorylation.

For phosphopepetide mapping, similar kinase assays were performed using 1 mM cold ATP (final concentration) and samples were alkylated with iodoacetamide [55]. Phosphopeptide mapping via LC/MS was carried out as described [55].

### Phylogenetic analyses

Putative orthologs of Cmb were identified by BlastP searches of GenBank as well as other arthropod genome repositories (see Fig. S1) using the sequence of the Cmb-RA isoform. To deduce the phylogenetic position of *cmb* among putative heretofore unidentified dipteran *cmb* orthologs (see Fig. S2A and B), a multiple sequence alignment of Cmb-RA against the deduced amino acid sequences of Dipteran orthologs was performed using T-Coffee (v6.85) [56], [57] using the following pair-wise alignment methods: the 10 best local alignments (Lalign_pair), an accurate global alignment (slow_pair) [56], [57]. This alignment was used to construct a maximum likelihood tree using the PhyML program (v3.0 aLRT) [58] using the WAG substitution model assuming an estimated proportion of invariant sites (of 0.018) and 4 gamma-distributed rate categories to account for rate heterogeneity across sites, with the gamma shape parameter being estimated directly from the data (gamma = 1.306) [59]. Internal branch support was assessed using the aLRT test (SH-Like).

A similar strategy was used to deduce the phylogenetic position of *cmb* among other identified putative arthropod orthologs (see Figs. S1 and S2C) using the WAG substitution model assuming an estimated proportion of invariant sites (of 0.007) and 4 gamma-distributed rate categories to account for rate heterogeneity across sites [59]. The gamma shape parameter was estimated directly from the data (gamma = 2.195). Reliability for internal branch was also assessed using the aLRT test (SH-Like). All phylogenetic analyses were performed using Phylogeny.fr [60]. All trees were edited and visualized using Geneious [61]. All alignments are available by request.

### Protein interactions

Two-hybrid assays were performed using the Clontech system according to the instructions of the manufacturer (see also [40]).

Co-immunoprecipitations were done with a modified version of the technique used in [41]. Briefly, HEK293 cells were transfected with 5 µg of pCS3_GFP_CmbRA, pCS3_GFP_CmbRB, pCS3_GFP_CmbInt or pCS_GFP_TBX1 together with 5 µg pCSMyc6_Mwh or pCS3_Myc_Dazap1 using Polyethylenimine (PEI). The cells were collected after 48 hours and washed with ice cold 1xPBS and lysed in in 500 ul of Buffer A (20 mM Tris, 100 mM NaCl, 1% Nonidet-P 40, 1 mM EDTA, 1 mM EGTA and 1∶200 concentration of 1 mM Benzamidine, 10 µM Leupeptin, and 1 µM Pepstatin) and rotated at 4°C for 15 minutes. Lysates were centrifuged at 1000 g for 15 minutes two times with the supernatant being retained after each step. The supernatants were incubated with 1 ug of mouse anti-GFP antibody (Roche) for four hours at 4°C. 30 ul of 50% Protein G beads were added and followed by overnight incubation at 4°C. Immunoprecipitates were washed 3x with cold Buffer A and suspended in 15 ul of 2x Laemmli buffer. Samples were run on a 12% SDS gel and western blot analysis was performed using standard protocols.

### Immunohistochemistry

Flip-out clones were generated using actin>stop>Gal4 with UAS-GFP as lineage tracer. 30 and 36 hpf Pupal wing discs were dissected and stained by following a standard procedure [20]. The discs were fixed in 4% Paraformaldehyde, washed with 1xPBS, incubated with the primary antibody overnight at 4°C, washed with 1xPBS/0.3% TritonX100, and incubated with a fluorescently labeled secondary antibody, washed and mounted in Prolong gold antifade reagent with DAPI (Invitrogen).

### Oligonucleotides

Fast_bac_his_upper GATCGCTGCAGCTGGATCCGGGAATTCACGCGGCTCCCATCACCATCACCATCACGGTTA

Fast_bac_his_lower AGCTTAACCGTGATGGTGATGGTGATGGGAGCCGCGTGAATTCCCGGATCCAGCTGCAGC

drok_Upper_BglII TATAGATCTATGCCAGCTGGACGAGAA

drok_lower_BamHI TATGGATCCTTTCAGCGATGAATTGGC

CG10732_left_for_KpnI TATGGTACCCGATGGGCCTTTGTTTGTAT

CG10732_left_rev_SacII ATACCGCGGGGGGCAAGAATTAAAGGATTT

CG10732_right_for_BglII TATAGATCTCGATGTTACGAGCGAACTGA

CG10732_rarmLong_rev_AvrII TATCCTAGGACCTCAGTGTGGACCTACCG

Cmb KO verification F CTGCAGGAGAAACTGC

Cmb KO verification R CTGACCTCGTCGTTGCTC

EY1065_1 TCACCGCCGATAACACTTGTG

EY1065_rev CACTGGCTCTGCCGTTCCACT

CG10732_RA_for TATAGATCTGCCACCATGGCGCCGCCGCCCAAG

CG10732_RA_Nrev TCGGTTGATATCTGCTATTCAGG

CG10732_RA_Cfor GAGGGAGCAGCTGGTGGA

CG10732_RA_rev ATATCTAGACTATTCGAGACTGACGTCCTG

CG10732 _RB_For TATGGATTCATGGTGCACGAAATCAAC

CG10732_RB_Rev CTCGAGTTACCTGCGGAAGTTGTC

T46A_10732F CTACACAGCGTCTTGGAAGCGCCCACGCCCGATACCACG

T46A_10732R CGTGGTATCGGGCGTGGGCGCTTCCAAGACGCTGTGTAG

T370AClu_10732F CTTGCCCCAGCACCCGCCCCCATGAGACAGCGG

T370AClu_10732R CCGCTGTCTCATGGGGGCGGGTGCTGGGGCAAG

S300A_10732F CGTAGTGAGGAAAGAACTGCCGTGGAGCGCCGAATTGCCG

S300A_10732R CGGCAATTCGGCGCTCCACGGCAGTTCTTTCCTCACTACG

T206A_10732F GAGAAAGAGGAGACTCCCGCGCCTCTTCCCAAGCCAGAG

T206A_10732R CTCTGGCTTGGGAAGAGGCGCGGGAGTCTCCTCTTTCTC

CG10732-NdeI-F1 GGGAATTCCATATG ATGGCGCCGCCGCCCAAG

CG10732-XmaI-R 1 TCCCCCCGGGATTTGAGGAGCCCCCTTG

CG10732-NdeI-F2 GGGAATTCCATATGGTGCCTGGAATGTGGGC

CG10732-XmaI-end-R2 TCCCCCCGGG CTATTCGAGACTGACGTCCTGTT

DBD-mwh5 GGAATTCCATATGGCTCCCAGTGTGTGCG

DBD-mwh3 CCGGAATTCTTAGTAGAGGCCGGATGGCAG

pGBKT7-5′Fy CATGCCATGGAGATGTCCATCTATTTGTTATG

pGBKT7-3′_Fy CGCGGATCCTTATCACCAACATACTGACTTC

5′frtz-hybrid GGGAATTCCATATGCTGCTCAGCGAGACC

3′fritz-hybrid CCGGAATTCTTATTAGACCACGCCGAAGTGGA

Inturn-th5 TCTAGGGAATTTCCATATGCGCAAATCGCCGGCCAG

Inturn-th3 GATCGCGGATCCATGTCATCCCATTGAGAAGAAGGA

## Supporting Information

Figure S1 **Summary and accession numbers of sequences used in alignments and phylogenetic analyses.**
(TIF)

Figure S2 **Cmb phylogenetic analyses.** (A) Maximum likelihood tree of the deduced amino acid sequence of *D. melanogaster* Cmb to other putative Dipteran Cmb proteins showing that Cmb is highly conserved within the Dipterans (including flies and mosquitos). Multiple, lineage-specific *cmb* duplications may have occurred in the mosquitoes, in that these mosquito *cmb* orthologs did not resolve into paralog-specific clades. However, the results of this analysis do suggest that the common ancestor of the *Anopheles* lineage had duplicate *cmb* paralogs with the most parsimonious explanation being that *A. darlingi* lost the paralog of *A. gambiae* AFAP012311. The relationships among other mosquito *cmb* genes is less clear and may be due long branch attraction [65]. (B) *Drosophila* portion of the tree in (A) showing the relationship of the deduced amino acid sequences of putative Drosophilid *cmb* orthologs. (C) Maximum likelihood tree showing the relationships between the deduced amino acid sequences of arthropod Cmb orthologs. A DELTA-BLAST of Cmb indicated that it has partial sequence similarity to SMC (Stability of Mitotic Chromosomes) proteins. The Cmb group clusters as a sister-group to the SMC2 protein group. aLRT test (SH-Like) support at each node is indicated as a percent. The scale bars represent the number of amino acid substitutions per site. All gene numbers were retrieved from the repositories listed in Figure S1.
(TIF)

Figure S3 **Multiple sequence alignment of the putative amino acid sequences of arthropod** ***Cmb*** **orthologs.** All aligned sequences are listed in Figure S1. Sequence logos are shown above each aligned site. Sequences were aligned using the T-COFFEE algorithm (see Materials and Methods).
(PNG)

Figure S4 **FASTA file of alignment shown in Fig. S3.**
(FASTA)

Figure S5 **Loss or gain of** ***cmb*** **does not cause a PCP phenotype in the eye.** Tangential sections of adult eyes with corresponding schematic representation of ommatidial orientations underneath. Black and red arrows represent dorsal and ventral chiral forms of ommatidia. (A) Wild-type. (B) A homozygous *cmb^KO^* mutant eye shows no PCP phenotype. (C, D) Eyes overexpressing *cmb-RA* (C) or *cmb-RB* (D) under the control of the *sev-Gal4* at 29°C are wild-type.
(TIF)

## References

[1] Devenport D, Fuchs E (2008) Planar polarization in embryonic epidermis orchestrates global asymmetric morphogenesis of hair follicles. Nat Cell Biol 10: 1257–1268. 18849982 10.1038/ncb1784 PMC2607065

[2] Guo N, Hawkins C, Nathans J (2004) From The Cover: Frizzled6 controls hair patterning in mice. Proc Natl Acad Sci U S A 101: 9277–9281. 15169958 10.1073/pnas.0402802101 PMC438967

[3] Montcouquiol M, Rachel RA, Lanford PJ, Copeland NG, Jenkins NA, et al (2003) Identification of Vangl2 and Scrb1 as planar polarity genes in mammals. Nature 423: 173–177. 12724779 10.1038/nature01618

[4] Gao B, Song H, Bishop K, Elliot G, Garrett L, et al (2011) Wnt signaling gradients establish planar cell polarity by inducing Vangl2 phosphorylation through Ror2. Dev Cell 20: 163–176. 21316585 10.1016/j.devcel.2011.01.001 PMC3062198

[5] Heisenberg CP, Tada M, Rauch GJ, Saude L, Concha ML, et al (2000) Silberblick/Wnt11 mediates convergent extension movements during zebrafish gastrulation. Nature 405: 76–81. 10811221 10.1038/35011068

[6] Wallingford JB, Rowning BA, Vogeli KM, Rothbacher U, Fraser SE, et al (2000) Dishevelled controls cell polarity during Xenopus gastrulation. Nature 405: 81–85. 10811222 10.1038/35011077

[7] Karner CM, Chirumamilla R, Aoki S, Igarashi P, Wallingford JB, et al (2009) Wnt9b signaling regulates planar cell polarity and kidney tubule morphogenesis. Nature genetics 41: 793–799. 19543268 10.1038/ng.400 PMC2761080

[8] Lienkamp SS, Liu K, Karner CM, Carroll TJ, Ronneberger O, et al (2012) Vertebrate kidney tubules elongate using a planar cell polarity-dependent, rosette-based mechanism of convergent extension. Nat Genet 44: 1382–1387. 23143599 10.1038/ng.2452 PMC4167614

[9] Gray RS, Roszko I, Solnica-Krezel L (2011) Planar cell polarity: coordinating morphogenetic cell behaviors with embryonic polarity. Developmental cell 21: 120–133. 21763613 10.1016/j.devcel.2011.06.011 PMC3166557

[10] Wallingford JB, Fraser SE, Harland RM (2002) Convergent extension: the molecular control of polarized cell movement during embryonic development. Dev Cell 2: 695–706. 12062082 10.1016/s1534-5807(02)00197-1

[11] Maung SM, Jenny A (2011) Planar cell polarity in Drosophila. Organogenesis 7: 165–179. 21983142 10.4161/org.7.3.18143 PMC3243030

[12] Strutt H, Strutt D (2009) Asymmetric localisation of planar polarity proteins: Mechanisms and consequences. Semin Cell Dev Biol 20: 957–963. 19751618 10.1016/j.semcdb.2009.03.006

[13] Vladar EK, Antic D, Axelrod JD (2009) Planar Cell Polarity Signaling: The Developing Cell’s Compass. Cold Spring Harbor Perspect Biol 1: a002964. 10.1101/cshperspect.a002964 PMC2773631 20066108

[14] Adler P (2005) Planar polarity in the *Drosophila* wing. In: Mlodzik M, editor. Planar cell polarization during development. San Diego: Elsevier. 1–14.

[15] Eaton S, Auvinen P, Luo L, Jan YN, Simons K (1995) CDC42 and Rac1 control different actin-dependent processes in the *Drosophila* wing disc epithelium. J Cell Biol 131: 151–164. 7559772 10.1083/jcb.131.1.151 PMC2120599

[16] Turner CM, Adler PN (1998) Distinct roles for the actin and microtubule cytoskeletons in the morphogenesis of epidermal hairs during wing development in *Drosophila* . Mech Dev 70: 181–192. 9510034 10.1016/s0925-4773(97)00194-9

[17] Gubb D, García-Bellido A (1982) A genetic analysis of the determination of cuticular polarity during development in *Drosophila melanogaster* . J Embryol Exp Morphol 68: 37–57. 6809878

[18] Wong LL, Adler PN (1993) Tissue polarity genes of Drosophila regulate the subcellular location for prehair initiation in pupal wing cells. The Journal of cell biology 123: 209–221. 8408199 10.1083/jcb.123.1.209 PMC2119819

[19] Guild GM, Connelly PS, Ruggiero L, Vranich KA, Tilney LG (2005) Actin filament bundles in Drosophila wing hairs: hairs and bristles use different strategies for assembly. Molecular biology of the cell 16: 3620–3631. 15917291 10.1091/mbc.E05-03-0185 PMC1182302

[20] Adler PN, Zhu C, Stone D (2004) Inturned localizes to the proximal side of wing cells under the instruction of upstream planar polarity proteins. Curr Biol 14: 2046–2051. 15556868 10.1016/j.cub.2004.11.007

[21] Strutt D, Warrington SJ (2008) Planar polarity genes in the Drosophila wing regulate the localisation of the FH3-domain protein Multiple Wing Hairs to control the site of hair production. Development 135: 3103–3111. 18701542 10.1242/dev.025205 PMC2556872

[22] Lu Q, Yan J, Adler PN (2010) The Drosophila planar polarity proteins inturned and multiple wing hairs interact physically and function together. Genetics 185: 549–558. 20351219 10.1534/genetics.110.114066 PMC2881136

[23] Yan J, Huen D, Morely T, Johnson G, Gubb D, et al (2008) The multiple-wing-hairs gene encodes a novel GBD-FH3 domain-containing protein that functions both prior to and after wing hair initiation. Genetics 180: 219–228. 18723886 10.1534/genetics.108.091314 PMC2535676

[24] Marlow F, Topczewski J, Sepich D, Solnica-Krezel L (2002) Zebrafish rho kinase 2 acts downstream of wnt11 to mediate cell polarity and effective convergence and extension movements. Curr Biol 12: 876–884. 12062050 10.1016/s0960-9822(02)00864-3

[25] Kim GH, Han JK (2005) JNK and ROKalpha function in the noncanonical Wnt/RhoA signaling pathway to regulate Xenopus convergent extension movements. Dev Dyn 232: 958–968. 15739222 10.1002/dvdy.20262

[26] Winter CG, Wang B, Ballew A, Royou A, Karess R, et al (2001) Drosophila Rho-associated kinase (Drok) links Frizzled-mediated planar cell polarity signaling to the actin cytoskeleton. Cell 105: 81–91. 11301004 10.1016/s0092-8674(01)00298-7

[27] Lee LA, Lee E, Anderson MA, Vardy L, Tahinci E, et al (2005) Drosophila genome-scale screen for PAN GU kinase substrates identifies Mat89Bb as a cell cycle regulator. Dev Cell 8: 435–442. 15737938 10.1016/j.devcel.2004.12.008

[28] Riechmann V, Ephrussi A (2004) Par-1 regulates bicoid mRNA localisation by phosphorylating Exuperantia. Development 131: 5897–5907. 15539486 10.1242/dev.01515

[29] St Pierre SE, Ponting L, Stefancsik R, McQuilton P, FlyBase C (2014) FlyBase 102–advanced approaches to interrogating FlyBase. Nucleic Acids Res 42: D780–788. 24234449 10.1093/nar/gkt1092 PMC3964969

[30] Hirano T (2002) The ABCs of SMC proteins: two-armed ATPases for chromosome condensation, cohesion, and repair. Genes Dev 16: 399–414. 11850403 10.1101/gad.955102

[31] Khandekar SS, Yi T, Dul E, Wright LL, Chen S, et al (2006) Expression, purification, and characterization of an enzymatically active truncated human rho-kinase I (ROCK I) domain expressed in Sf-9 insect cells. Protein Pept Lett 13: 369–376. 16712513 10.2174/092986606775974357

[32] Amano M, Ito M, Kimura K, Fukata Y, Chihara K, et al (1996) Phosphorylation and activation of myosin by Rho-associated kinase (Rho-kinase). J Biol Chem 271: 20246–20249. 8702756 10.1074/jbc.271.34.20246

[33] Fukata Y, Oshiro N, Kinoshita N, Kawano Y, Matsuoka Y, et al (1999) Phosphorylation of adducin by Rho-kinase plays a crucial role in cell motility. J Cell Biol 145: 347–361. 10209029 10.1083/jcb.145.2.347 PMC2133101

[34] Goto H, Kosako H, Tanabe K, Yanagida M, Sakurai M, et al (1998) Phosphorylation of vimentin by Rho-associated kinase at a unique amino-terminal site that is specifically phosphorylated during cytokinesis. J Biol Chem 273: 11728–11736. 9565595 10.1074/jbc.273.19.11728

[35] Kang JH, Asai D, Tsuchiya A, Mori T, Niidome T, et al (2011) Peptide substrates for Rho-associated kinase 2 (Rho-kinase 2/ROCK2). PLoS One 6: e22699. 21818369 10.1371/journal.pone.0022699 PMC3144920

[36] Matsui T, Maeda M, Doi Y, Yonemura S, Amano M, et al (1998) Rho-kinase phosphorylates COOH-terminal threonines of ezrin/radixin/moesin (ERM) proteins and regulates their head-to-tail association. J Cell Biol 140: 647–657. 9456324 10.1083/jcb.140.3.647 PMC2140160

[37] Huang J, Zhou W, Watson AM, Jan YN, Hong Y (2008) Efficient ends-out gene targeting in Drosophila. Genetics 180: 703–707. 18757917 10.1534/genetics.108.090563 PMC2535722

[38] Rong YS, Golic KG (2000) Gene targeting by homologous recombination in Drosophila. Science 288: 2013–2018. 10856208 10.1126/science.288.5473.2013

[39] Boutros M, Mlodzik M (1999) Dishevelled: at the crossroads of divergent intracellular signaling pathways. Mech Dev 83: 27–37. 10507837 10.1016/s0925-4773(99)00046-5

[40] Jenny A, Darken RS, Wilson PA, Mlodzik M (2003) Prickle and Strabismus form a functional complex to generate a correct axis during planar cell polarity signaling. Embo J 22: 4409–4420. 12941693 10.1093/emboj/cdg424 PMC202366

[41] Jenny A, Reynolds-Kenneally J, Das G, Burnett M, Mlodzik M (2005) Diego and Prickle regulate Frizzled planar cell polarity signalling by competing for Dishevelled binding. Nat Cell Biol 7: 691–697. 15937478 10.1038/ncb1271

[42] Yan J, Lu Q, Fang X, Adler PN (2009) Rho1 has multiple functions in Drosophila wing planar polarity. Developmental biology 333: 186–199. 19576201 10.1016/j.ydbio.2009.06.027 PMC2728161

[43] Amano M, Nakayama M, Kaibuchi K (2010) Rho-kinase/ROCK: A key regulator of the cytoskeleton and cell polarity. Cytoskeleton (Hoboken) 67: 545–554. 20803696 10.1002/cm.20472 PMC3038199

[44] Taylor SS, Kornev AP (2011) Protein kinases: evolution of dynamic regulatory proteins. Trends Biochem Sci 36: 65–77. 20971646 10.1016/j.tibs.2010.09.006 PMC3084033

[45] Leung T, Chen XQ, Manser E, Lim L (1996) The p160 RhoA-binding kinase ROK alpha is a member of a kinase family and is involved in the reorganization of the cytoskeleton. Mol Cell Biol 16: 5313–5327. 8816443 10.1128/mcb.16.10.5313 PMC231530

[46] Davis RJ (1993) The mitogen-activated protein kinase signal transduction pathway. J Biol Chem 268: 14553–14556. 8325833

[47] Collier S LH, Burgess R, Adler P (2005) The WD40 repeat protein fritz links cytoskeletal planar polarity to frizzled subcellular localization in the Drosophila epidermis. Genetics 169: 2035–2045. 15654087 10.1534/genetics.104.033381 PMC1449578

[48] Dunlop JA (2010) Geological history and phylogeny of Chelicerata. Arthropod Struct Dev 39: 124–142. 20093195 10.1016/j.asd.2010.01.003

[49] Dickinson WJ, Thatcher JW (1997) Morphogenesis of denticles and hairs in Drosophila embryos: involvement of actin-associated proteins that also affect adult structures. Cell Motil Cytoskeleton 38: 9–21. 9295137 10.1002/(SICI)1097-0169(1997)38:1<9::AID-CM2>3.0.CO;2-4

[50] Lee H, Adler PN (2002) The function of the frizzled pathway in the Drosophila wing is dependent on inturned and fuzzy. Genetics 160: 1535–1547. 11973308 10.1093/genetics/160.4.1535 PMC1462037

[51] Yun UJ, Kim SY, Liu J, Adler PN, Bae E, et al (1999) The inturned protein of Drosophila melanogaster is a cytoplasmic protein located at the cell periphery in wing cells. Dev Genet 25: 297–305. 10570461 10.1002/(SICI)1520-6408(1999)25:4<297::AID-DVG3>3.0.CO;2-L

[52] Collier S, Gubb D (1997) Drosophila tissue polarity requires the cell-autonomous activity of the fuzzy gene, which encodes a novel transmembrane protein. Development 124: 4029–4037. 9374400 10.1242/dev.124.20.4029

[53] Katzen F (2007) Gateway((R)) recombinational cloning: a biological operating system. Expert Opin Drug Discov 2: 571–589. 23484762 10.1517/17460441.2.4.571

[54] Villefranc JA, Amigo J, Lawson ND (2007) Gateway compatible vectors for analysis of gene function in the zebrafish. Dev Dyn 236: 3077–3087. 17948311 10.1002/dvdy.21354 PMC4518551

[55] Schlosser A, Vanselow JT, Kramer A (2005) Mapping of phosphorylation sites by a multi-protease approach with specific phosphopeptide enrichment and NanoLC-MS/MS analysis. Anal Chem 77: 5243–5250. 16097765 10.1021/ac050232m

[56] Notredame C, Higgins DG, Heringa J (2000) T-Coffee: A novel method for fast and accurate multiple sequence alignment. Journal of Molecular Biology 302: 205–217. 10964570 10.1006/jmbi.2000.4042

[57] Notredame C, Higgins DG, Heringa J (2000) T-Coffee: A novel method for fast and accurate multiple sequence alignment. J Mol Biol 302: 205–217. 10964570 10.1006/jmbi.2000.4042

[58] Guindon S, Lethiec F, Duroux P, Gascuel O (2005) PHYML Online–a web server for fast maximum likelihood-based phylogenetic inference. Nucleic Acids Res 33: W557–559. 15980534 10.1093/nar/gki352 PMC1160113

[59] Whelan S, Goldman N (2001) A general empirical model of protein evolution derived from multiple protein families using a maximum-likelihood approach. Mol Biol Evol 18: 691–699. 11319253 10.1093/oxfordjournals.molbev.a003851

[60] Dereeper A, Guignon V, Blanc G, Audic S, Buffet S, et al (2008) Phylogeny.fr: robust phylogenetic analysis for the non-specialist. Nucleic Acids Res 36: W465–469. 18424797 10.1093/nar/gkn180 PMC2447785

[61] Kearse M, Moir R, Wilson A, Stones-Havas S, Cheung M, et al (2012) Geneious Basic: an integrated and extendable desktop software platform for the organization and analysis of sequence data. Bioinformatics 28: 1647–1649. 22543367 10.1093/bioinformatics/bts199 PMC3371832

[62] Regier JC, Mitter C, Zwick A, Bazinet AL, Cummings MP, et al (2013) A large-scale, higher-level, molecular phylogenetic study of the insect order Lepidoptera (moths and butterflies). PLoS One 8: e58568. 23554903 10.1371/journal.pone.0058568 PMC3595289

[63] Regier JC, Shultz JW, Zwick A, Hussey A, Ball B, et al (2010) Arthropod relationships revealed by phylogenomic analysis of nuclear protein-coding sequences. Nature 463: 1079–1083. 20147900 10.1038/nature08742

[64] Wan X, Kim MI, Kim MJ, Kim I (2012) Complete mitochondrial genome of the free-living earwig, Challia fletcheri (Dermaptera: Pygidicranidae) and phylogeny of Polyneoptera. PLoS One 7: e42056. 22879905 10.1371/journal.pone.0042056 PMC3412835

[65] Philippe H, Zhou Y, Brinkmann H, Rodrigue N, Delsuc F (2005) Heterotachy and long-branch attraction in phylogenetics. BMC Evol Biol 5: 50. 16209710 10.1186/1471-2148-5-50 PMC1274308

