# Supplementary figures and images for: Combover/CG10732, a Novel PCP Effector for Drosophila Wing Hair Formation
Source: PLoS One. 2014 Sep 10;9(9):e107311. doi: 10.1371/journal.pone.0107311 (PMC4160248; doi:10.1371/journal.pone.0107311)

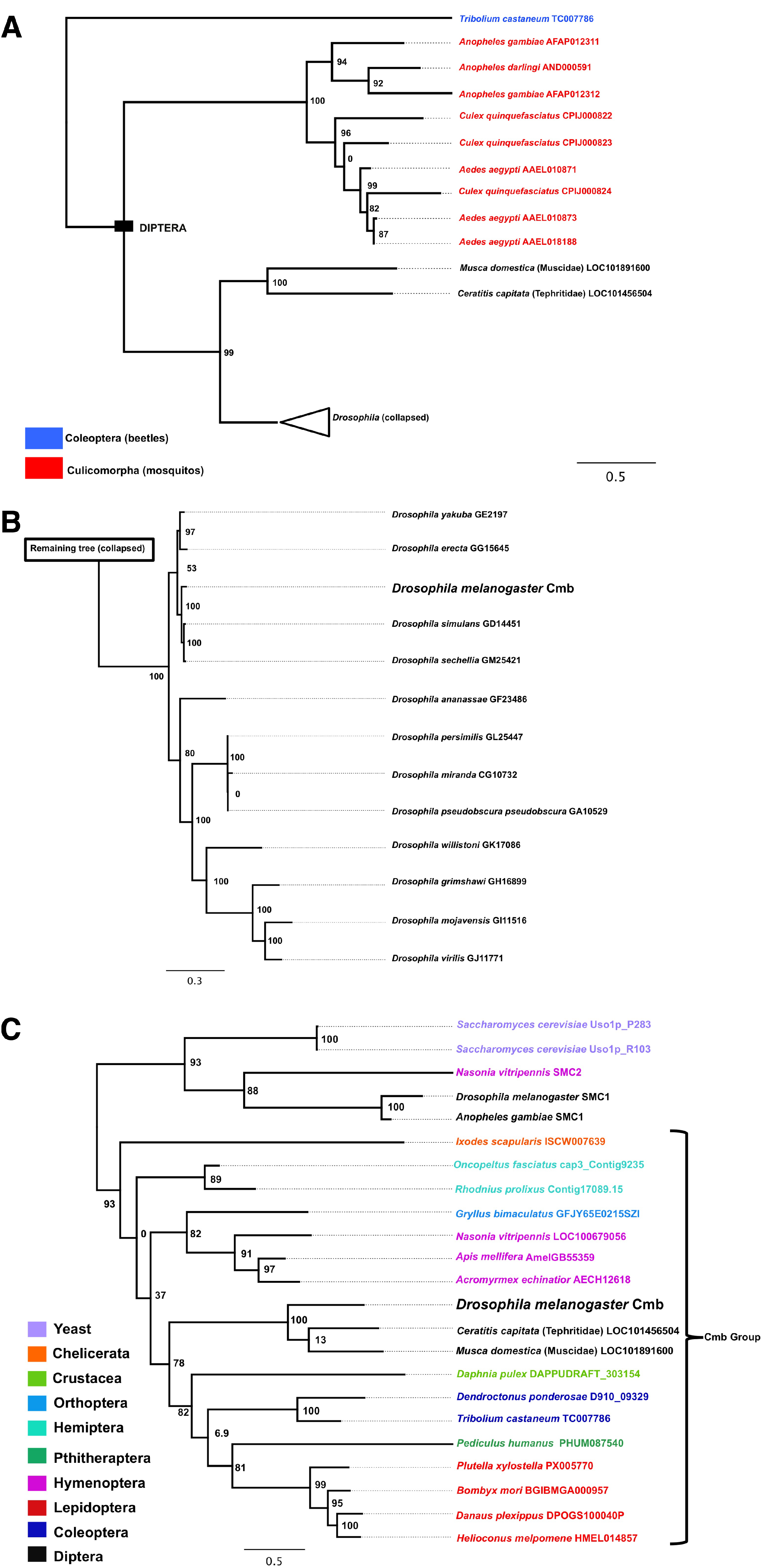

Supplement: Figure S1 — Summary and accession numbers of sequences used in alignments and phylogenetic analyses. (TIF) [file pone.0107311.s001.tif]

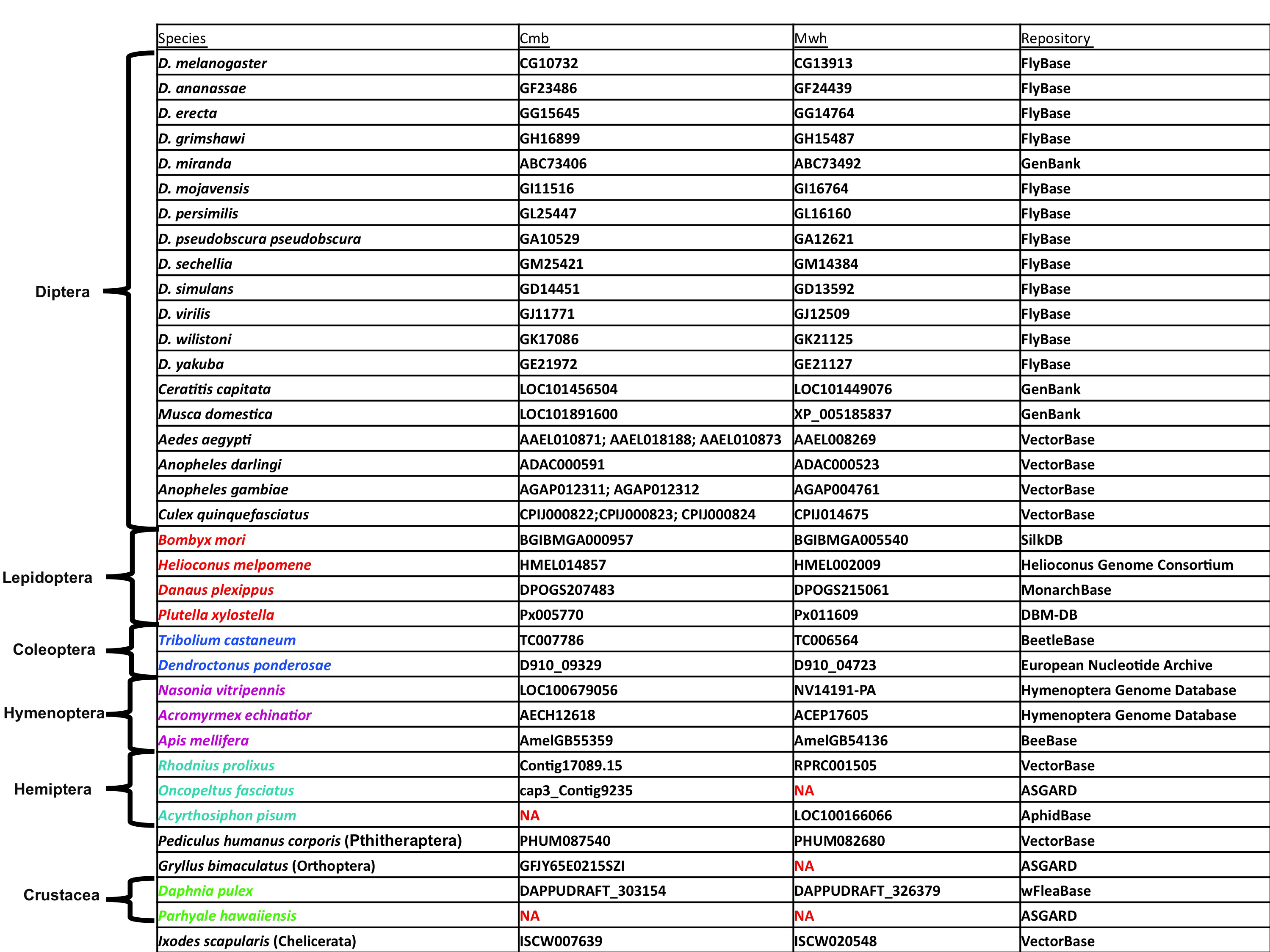

Supplement: Figure S2 — Cmb phylogenetic analyses. (A) Maximum likelihood tree of the deduced amino acid sequence of D. melanogaster Cmb to other putative Dipteran Cmb proteins showing that Cmb is highly conserved within the Dipterans (including flies and mosquitos). Multiple, lineage-specific cmb duplications may have occurred in the mosquitoes, in that these mosquito cmb orthologs did not resolve into paralog-specific clades. However, the results of this analysis do suggest that the common ancestor of the Anopheles lineage had duplicate cmb paralogs with the most parsimonious explanation being that A. darlingi lost the paralog of A. gambiae AFAP012311. The relationships among other mosquito cmb genes is less clear and may be due long branch attraction [65]. (B) Drosophila portion of the tree in (A) showing the relationship of the deduced amino acid sequences of putative Drosophilid cmb orthologs. (C) Maximum likelihood tree showing the relationships between the deduced amino acid sequences of arthropod Cmb orthologs. A DELTA-BLAST of Cmb indicated that it has partial sequence similarity to SMC (Stability of Mitotic Chromosomes) proteins. The Cmb group clusters as a sister-group to the SMC2 protein group. aLRT test (SH-Like) support at each node is indicated as a percent. The scale bars represent the number of amino acid substitutions per site. All gene numbers were retrieved from the repositories listed in Figure S1. (TIF) [file pone.0107311.s002.tif]

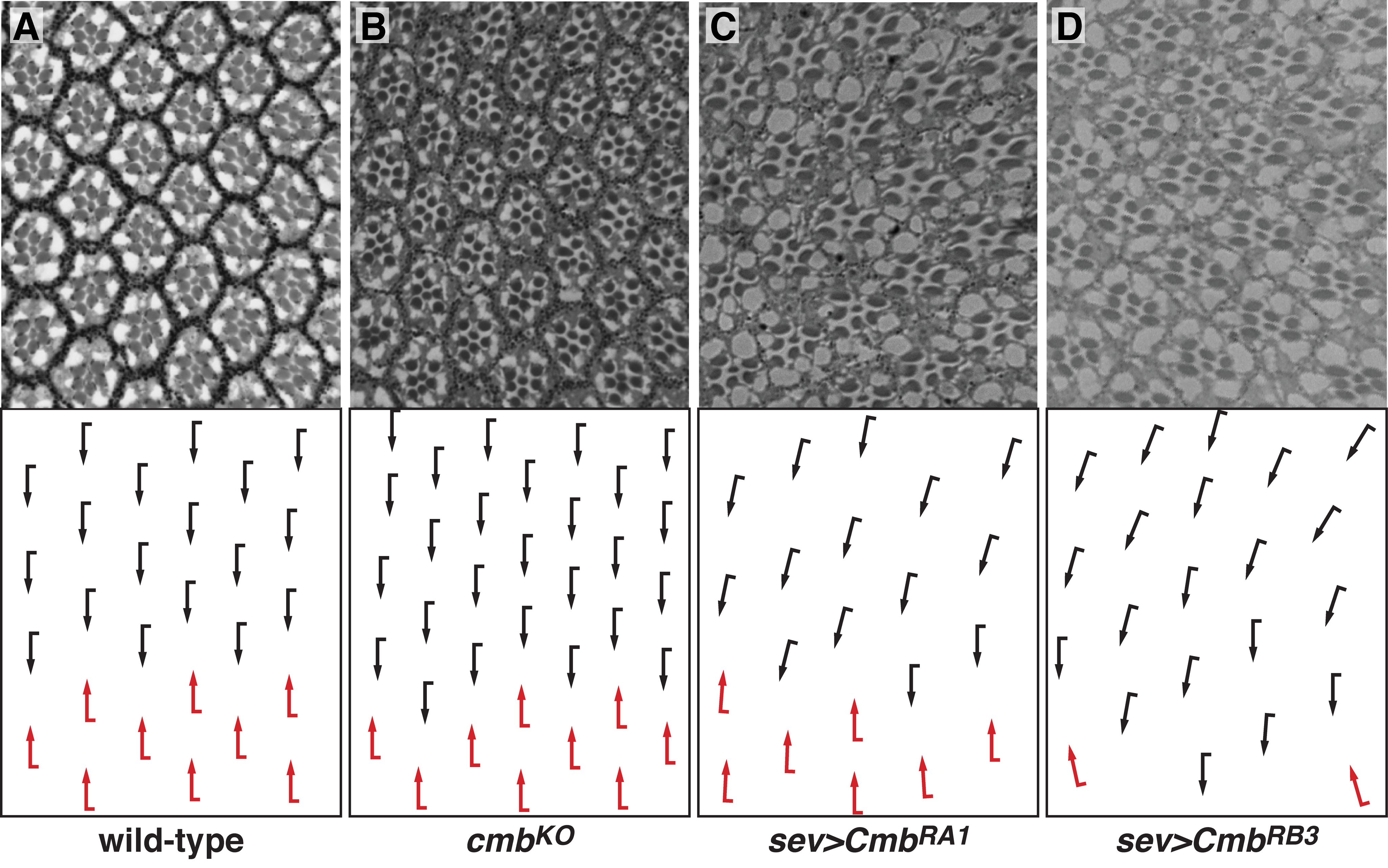

Supplement: Figure S5 — Loss or gain of cmb does not cause a PCP phenotype in the eye. Tangential sections of adult eyes with corresponding schematic representation of ommatidial orientations underneath. Black and red arrows represent dorsal and ventral chiral forms of ommatidia. (A) Wild-type. (B) A homozygous cmbKO mutant eye shows no PCP phenotype. (C, D) Eyes overexpressing cmb-RA (C) or cmb-RB (D) under the control of the sev-Gal4 at 29°C are wild-type. (TIF) [file pone.0107311.s005.tif]
